# Cancer-associated fibroblasts from human NSCLC survive ablative doses of radiation but their invasive capacity is reduced

**DOI:** 10.1186/1748-717X-7-59

**Published:** 2012-04-13

**Authors:** Turid Hellevik, Ingvild Pettersen, Vivian Berg, Jan Olof Winberg, Bjørn T Moe, Kristian Bartnes, Ruth H Paulssen, Lill-Tove Busund, Roy Bremnes, Anthony Chalmers, Iñigo Martinez-Zubiaurre

**Affiliations:** 1 Department of Oncology, University Hospital of Northern Norway, 9038 Tromsø, Norway; 2 Department of Clinical Medicine, University of Tromsø, 9037, Tromsø, Norway; 3 Department of Medical Biology, University of Tromsø, 9037 Tromsø, Norway; 4 Department of Cardiothoracic and Vascular Surgery, University Hospital of Northern Norway, 9038 Tromsø, Norway; 5 Department of Clinical Pathology, University Hospital of Northern Norway, 9038 Tromsø, Norway; 6 Institute of Cancer Sciences, University of Glasgow, Glasgow, UK

**Keywords:** Cancer-associated fibroblasts, Ablative radiation, Invasion, Integrins, Focal adhesion

## Abstract

**Background:**

Cancer-Associated Fibroblasts (CAFs) are significant components of solid malignancies and play central roles in cancer sustainability, invasion and metastasis. In this study we have investigated the invasive capacity and matrix remodelling properties of human lung CAFs after exposure to ablative doses of ionizing radiation (AIR), equivalent to single fractions delivered by stereotactic ablative radiotherapy (SART) for medically inoperable stage-I/II non-small-cell lung cancers.

**Methods:**

CAFs were isolated from lung tumour specimens from 16 donors. Initially, intrinsic radiosensitivity was evaluated by checking viability and extent of DNA-damage response (DDR) at different radiation doses. The migrative and invasive capacities of CAFs were thereafter determined after a sub-lethal single radiation dose of 18 Gy. To ascertain the mechanisms behind the altered invasive capacity of cells, expression of matrix metalloproteinases (MMPs) and their endogenous inhibitors (TIMPs) were measured in the conditioned media several days post-irradiation, along with expression of cell surface integrins and dynamics of focal contacts by vinculin-staining.

**Results:**

Exposing CAFs to 1 × 18 Gy resulted in a potent induction of multiple nuclear DDR foci (> 9/cell) with little resolution after 120 h, induced premature cellular senescence and inhibition of the proliferative, migrative and invasive capacity. AIR promoted MMP-3 and inhibited MMP-1 appearance to some extent, but did not affect expression of other major MMPs. Furthermore, surface expression of integrins α2, β1 and α5 was consistently enhanced, and a dramatic augmentation and redistribution of focal contacts was observed.

**Conclusions:**

Our data indicate that ablative doses of radiation exert advantageous inhibitory effects on the proliferative, migratory and invasive capacity of lung CAFs. The reduced motility of irradiated CAFs might be a consequence of stabilized focal contacts via integrins.

## Background

Stereotactic ablative radiotherapy (SART), or stereotactic body radiotherapy (SBRT), represents a novel technique with particular impact on medically inoperable stage I non-small-cell lung cancers (NSCLC) [1,2]. The enhanced accuracy offered by SART allows for delivery of high (or ablative) doses of ionizing radiation (IR) in oligofractionated regimens, resulting in remarkable tumour control with minimal toxicity [3]. Despite these encouraging clinical results, our knowledge of the radiobiological mechanisms associated with ablative radiotherapy (RT) is still limited.

There is increasing awareness that solid malignancies do not only contain transformed neoplastic cells, but are rather composed of a mixed population of cells and extracellular matrix that collectively constitute the tumour microenvironment, also known as the tumour stroma [4]. Reactive fibroblasts are frequently found in the stroma of human carcinomas, and their presence in large numbers is associated with high-grade malignancy and poor prognosis. Among multiple functions that contribute to tumorigenesis, CAFs are active providers of collagens, fibronectins, laminin, tenascin and proteoglycans, as well as ECM-degrading enzymes such as MMPs, cathepsins and plasminogen activator [5,6]. Stromal fibroblasts have also been shown to play a key role in the process of invasion by "paving the path" for tumour cells [7] or serving as initiators and stabilisers of tumour vessels [8]. Hence, by migrating and degrading matrix, CAFs make a direct contribution to tumour cell invasion, tumour vessel formation, and tumour growth [9].

It is evident that therapeutic irradiation of tumours will inevitably affect the total tumour stroma. Despite this undeniable fact; only limited knowledge is available regarding the responses of reactive fibroblasts to radiation. The importance of CAFs in the context of radiation has been revealed by others, thus recent reports indicate that fibroblasts of the pancreas may exert radioprotective effects over the malignant counterparts [10]. Overall, very few studies have been conducted with freshly isolated fibroblast from human tumour specimens [11,12]. Previous reports using cell lines have shown that after relatively high radiation doses, fibroblasts develop a senescent phenotype over several days with a concomitant and permanent DNA damage response, and acquire a pro-tumorigenic phenotype that favours tumour development through the release of paracrine signals [13-15]. In the context of SART, large individual radiation doses may have "ablative" effects on malignant cells but tumour stromal fibroblasts, which are relatively radioresistant, may survive the radiation insult. Hence, the ultimate effects of such large individual doses may be even more dependent on stromal components than conventional fractionated radiotherapy [16-18]. The aim of this study was to investigate the impact of ablative doses of ionizing radiation on CAFs freshly isolated from human lung cancers (NSCLCs), focusing on their migratory and matrix remodelling properties.

## Methods

### Human material, cell isolation and CAF cultures

Human CAFs were harvested from freshly resected non-small cell lung carcinoma (NSCLC) tumour tissues. Tumours from 16 patients were included in this study (Table 1). The Regional Ethical Committee approved the study, and all patients provided written informed consent. Fibroblasts from tumours were isolated using the out-growth method and characterized by specific antibodies. Briefly, tumour resections were collected and cut into 1-1.5 mm^3 ^pieces. Enzymatic digestion of tissues was carried out for 1.5 h with collagenase (Cat. no. C-9407 Sigma-Aldrich, St. Louise, MO, USA), at a final concentration of 0.8 mg/mL. Pure fibroblast cultures were obtained by selective cell detachment from the primary culture mix, and by further cell propagation in the presence of 10% FBS. Cells were grown at 3% oxygen levels and used for experiments after the second passage (2-3 weeks). Antibodies: FITC-conjugated anti-human α-SMA (smooth muscle α-actin) antibody (Abcam; Cat. # ab8211), FITC-conjugated anti-IgG antibody (negative control) and anti-human FAP (Fibroblast Activation Protein) α-antibody (Abcam; Cat. # ab53066).

**Table 1:** Donor features corresponding to the CAF cell lines used in this study

Donors	Age (years)	Sex	Tumourtype	T-size (mm)	T-stage
1	71	M	BAA	20	1a

2	72	F	BAA	16	1a

3	70	M	AC	23	1b

4	72	F	SCC	32	2b

5	63	M	AC	30	1b

6	78	M	AC	38	2b

7	78	M	SCC	20	1a

8	48	F	AC	13	1a

9	69	F	AC	30	1b

10	73	M	SCC	18	1a

11	64	F	AC	8	1a

12	50	M	LCC	50	2a

13	64	M	AC	7	1a

14	51	M	AC	60	2a

15	72	F	SCC	40	2a

16	73	M	AC	11	1a

Abbreviations: *BAA = bronchio alveolar adenocarcinoma; AC = adenocarcinoma*

SCC = Squamous cell carcinoma; LCC = Large cell carcinoma; M = male; F = female

### Irradiation of cells

Radiation protocols were established after initial dose-escalating pilot trials, and by comparing single dose with fractionated schedules. Hence, adherent CAFs were irradiated with high energy photons produced by a Varian clinical linear-accelerator, delivered as single doses of 2, 6, 12 and 18 Gy or as 6 × 3 Gy in 24-h intervals. Standard parameters for dose delivery was depth 30 mm, beam quality 15 MV, dose-rate 6 Gy/min and field size 20 × 20 cm. Radiation-doses were confirmed to be correct within an acceptable ± 4% by Thermo-Luminescent Dosimeters (TLDs). Cell survival/death after radiation was assessed by checking the extent of cell detachment by light microscopy during the following three weeks. Standard assays to test viability, such as MTT and "Clonogenic assay" could not be used in our system since the differences observed after long incubation periods between irradiated and non-irradiated cells were a consequence of premature cell senescence rather than cell death. Of note, in our internal control experiments all cells were able to exclude trypan blue, and no cell detachment was observed over three weeks in culture post-irradiation.

### Immunofluorescence staining

CAFs were cultured in 2-well chamber slides (Nunc, Thermo Fisher Scientific, NY, USA), fixed with 4% PFA-PBS for 10 min and permeabilized with 0.2% Triton-PBS for 8 min. Slides were then exposed to blocking buffer (2% HSA-PBS). Next; primary antibodies (Rabbit anti human 53BP1; Cat.#ab36823, or monoclonal anti-human Vinculin, Cat.#ab11194, Abcam Cambridge, UK) were diluted in blocking buffer and incubated with CAFs for 45 min at RT. After washing, cells were incubated with secondary antibody (anti-rabbit-Alexa546, Cat. #A11010, or anti-mouse-Alexa488, Cat. #A11110 Molecular Probes/Invitrogen, Leiden, The Netherlands) in blocking buffer, 30 min at RT. A second wash was followed by preparation of slides in DAPI-Fluoromount-G (Cat. # 0100-20, Southern Biotech, Birmingham, AL, USA). Specimens were examined in a fluorescence microscope (Zeiss Axiophot, Germany) equipped with a Nikon DS-5MC digital camera, and images were processed with Adobe^® ^Photoshop Software (CS5).

### Real-time monitoring of density dependent growth

To monitor cellular adhesion and growth responses we have exploited the "xCELLigence" system from Roche Applied Sciences (Indianapolis, IN), consisting of microtiter plates (E-plates) with integrated gold microarrays in the bottom of wells for continuous and label-free measurements of cellular status in real-time by the RTCA-DP instrument. Cell status is measured by electrical impedance and the relative change between impedance measured at any time (t) and baseline at time zero (t_0_) is displayed as the dimensionless parameter "Cell Index" (CI). In standard E-plates, CI-values are proportional to number of cells attached, and the kinetic profiles generated thus reflect adhesion and spreading within the first ~6 h upon seeding and thereafter mirror cell growth (increasing CI) and/or cell death (decreasing CI) [19-21]. In our study, control and irradiated CAFs from four randomly selected donors were brought into suspension and seeded in E-plates at a density of 6000 cells/well. E-plates were then transferred to the RTCA-DP instrument for automated real-time monitoring at standard incubator conditions, with quadruplet read-outs of the parameter "Cell Index" every 30 min the following 7 days.

### Real-time monitoring of cellular migration and invasion

Onset and rate of migration and invasion was also monitored by the "xCELLigence-system" as explained above, but using CIM (Cell Invasion-and-Migration)-plates rather than E-plates. CIM-plates feature microelectronic sensors located on the underside of a microporous membrane insert [22]. Cells capable of migrating from the upper chamber through the membrane and into the bottom chamber will contact and adhere to the sensors, resulting in increased impedance and hence increased "Cell Index" read-outs. Our migration assays were performed by seeding control and irradiated CAFs in the upper chamber of CIM-plates in serum-free medium and at a density of 50,000 cells per well. Bottom chambers of the CIM-plates were filled with serum-containing medium to promote migration across membranes towards the serum gradient. After seeding, CIM-plates were transferred into the RTCA DP instrument for continuous read-outs during 48 h. Impedance (i.e. "Cell index") was registered only from cells capable of migrating through the 8 μm porous membranes, and was performed in triplicates. For invasion assays, protocols identical to that for migration were followed, with the exception that upper chambers were loaded with 30 μL of a 1:10 dilution of Matrigel to create a 3D biomatrix film in each well prior to cell loading. For comparison, each pull of non-irradiated CAFs was also seeded in duplicates on Matrigel-free wells, reflecting pure migratory behaviour.

### β-galactosidase assays

CAFs were seeded at a density of 20,000 cells per well in 6-well plates and left for attachment and spreading for 24 h prior to irradiation. Five days post-irradiation cultures were washed and fixed for 5-7 min at RT with PFA (2%). β-galactosidase (5-bromo-4chloro-3-indolyl-B-D-galactopyranoside) staining was achieved following instructions from the manufacturer; "Senescence Cells Histochemical Staining Kit" (Cat.no CS0030, Sigma-Aldrich, St. Louise, MO, USA). Number of β-galactosidase active and senescent cells was determined by counting blue cells on 3 randomly selected fields under a Nikon Eclipse TS100 model light microscope. Randomly selected fields were photographed at 1000× magnification, using an Idea SPOT digital camera.

### Fluorescent bead-based fluorokine-multi analytes profiling assay (Luminex)

Quantitative measurements of MMPs and TIMPs were performed using a suspension array technique (Bio-plex 200, Bio-Rad, CA, USA). Five days post-irradiation CAF culture medium from five randomly selected donors was conditioned for 24 h. Protein levels of MMPs (MMP-1, -2, -3, -7, -8, -9, and -13) were analysed with a MMP multiplex kit (Cat. No. LMP-000, R&D Systems, MI, USA), whereas levels of TIMPs (TIMP-1, -2, -3 and -4) were analysed with a TIMP multiplex kit (Cat. No. LKT-003, R&D Systems). Samples were run in duplicates, and in dilutions 1:5 (MMPs) or 1:10 (TIMPs). Levels of MMPs and TIMPs were detected using the Bio-plex 200 analyser, according to instructions from the manufacturer. Data were analysed using SPSS statistical software version 16.0 (SPSS Inc., Chicago, IL, USA). Secretion of MMPs and TIMPS were examined for statistical significance using the Wilcoxon signed-rank test. All data are expressed as mean ± standard error of the mean (SEM). A p-value < 0.05 denoted the presence of a statistically significant difference.

### Flow cytometry

Adherent cultures of CAFs were grown in 6-well plates to 50% confluence and then irradiated, followed by incubation for another 5 days. Irradiated and control cells were detached by exposure to EDTA-PBS (2 mM), and fixed in cold 2%PFA-PBS. After fixation cells were kept in blocking solution (HSA-PBS) followed by exposure to FITC-conjugated anti-integrin antibodies (α2, a5 and β1, Cat.# ab30486, ab78043, ab46920 respectively, Abcam, Cambridge, UK) for 1 h at 22°C. Unlabeled antibodies were eliminated by a series of cell washings. CAFs incubated with FITC-conjugated IgG antibodies represented negative controls. Surface binding of primary antibodies was quantified on a Becton Dickinson FACScan flow cytometer. Cellular profiles were gated on intact cells, and were based on morphology and mean fluorescent intensity.

### Western blots

Six days post-irradiation attached cells were lysed in 500 μl RIPA lysis buffer (cat. no. 20-188, Millipore) containing protease inhibitors (cat. no. 20-201, Millipore). Protein content was measured by the Bradford assay (Bio-Rad Laboratories, CA, USA). Equal amounts of protein per sample were run in NuPAGE, Novex and Tris-Acetat Mini Gels (Invitrogen, Carlsbad, CA, USA) 4-12% by reduced conditions, and proteins were transferred to a PVDF (Pierce, Rockford, IL, USA) membrane and incubated with primary antibodies at 4°C overnight. Protein binding was visualized by the application of a secondary antibody, i.e. HRP-conjugated goat anti-rabbit IgG antibody (Cat.#: AP307P; Millipore).

## Results

### Isolation, characterization and purity of CAFs

**Figure 1:**
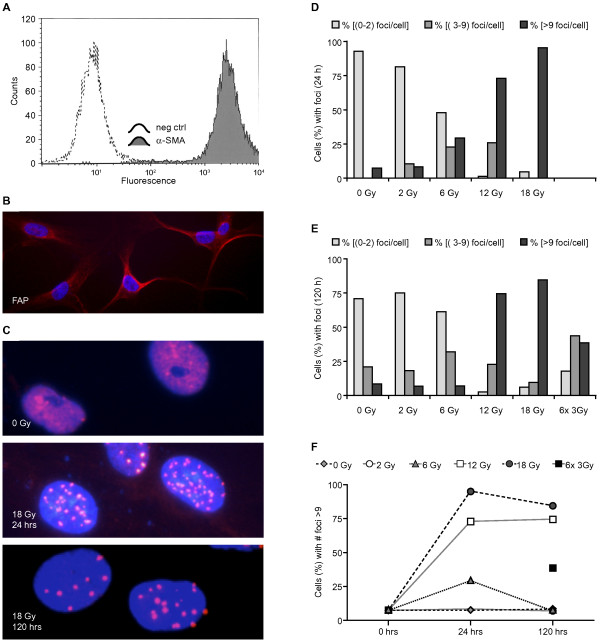
**Characterization of cultures and dynamics of 53BP-1 nuclear foci formation in dose-escalating IR exposures**: In **(A)**, flow cytometry of CAFs after staining cells with α-SMA antibody. Panel (**B**) shows immunostaining of CAFs with α-FAP. Fluorescence micrographs in (**C**) show staining with anti-53BP-1 (red/pink) and DAPI (blue). Induction of DNA damage response (DDR) and extent of repair was determined by counting percentage of cells showing nuclear foci staining of 53BP-1 at early (24 h; **D**) and late (5 days; **E**) time points. Columns in (**D**) and (**E**) each represent average counts from two randomly selected donors (n = 2) (donors #15 and #16). In panel (**F**), percentage cells with multi-foci (> 9/cell) in (**D**) and **(E)** are plotted versus time.

All the data generated in this study comes from cells directly prepared from lung tumour specimens obtained from different donors after surgical resection (Table 1). Tumour stromal fibroblasts were isolated by the outgrowth method, purified by selective cell detachment and expanded in serum-containing medium. Cells were ready for experimentation three weeks after tissue collection. Cell authentication and purity was checked by flow cytometry of cells exposed to the fibroblast specific marker smooth muscle α-actin (α-SMA) (Figure 1A). Analyses showed a single cell population 100% positive for α-SMA. Additionally, the reactive nature of the fibroblasts was checked by specific immuno staining with anti-fibroblast activation protein (FAP) antibody [23,24] (Figure 1B). All adherent cells in the cultures had a strong immuno-reactivity for FAP.

### Cellular responses to dose-escalating radiation exposures

**Figure 2:**
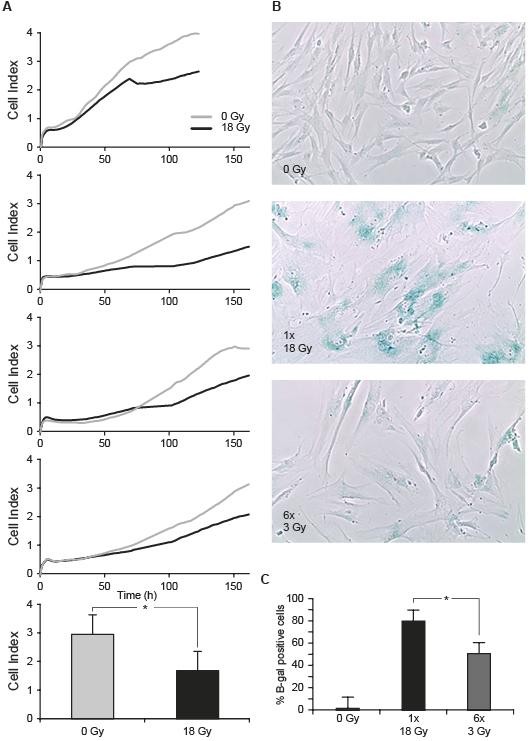
**Attenuation of the proliferative capacity of irradiated CAFs, and induction of premature senescence: (A) **Proliferation curves monitored over one week of non-irradiated cells (grey line) and cells receiving 1 × 18 Gy (black line) from four randomly selected donors (donors #15, #6, #7, #8 in Table 1). In lower panel, statistically significant differences found after applying paired sample *t-test *on values at the end point of the experiments are marked with * (p < 0.05). **(B) **β-galactosidase staining assay in CAFs from one randomly selected donor. The percentage of β-galactosidase positive cells in each condition was calculated from three different donors (donors #1, #7, #11 respectively from Table 1) and plotted in (**C**). Statistically significant differences are marked with * (p < 0.05).

Initial experiments were conducted to test the intrinsic radiosensitivity of CAFs by examining viability and DNA damage responses after different radiation doses. CAF cultures from two different donors were exposed to single radiation doses of 2, 6, 12 or 18 Gy and a fractionated regimen of 6 × 3 Gy. Induction and repair of DNA damage was determined by quantifying 53BP-1 containing nuclear foci, 24 h and 5 days post-irradiation (Figure 1C-F). Of note, no cell death was observed after any of the radiation regimens over a 3 weeks period, assessed by light microscopy (data not shown). Additionally, no reduction in cell viability was registered by "xCELLigence" during the first week post-irradiation (18 Gy) (Figure 2A). After low doses of radiation (2 Gy), resolution of 53BP-1 foci was observed over 24 h as expected (Figure 1D). After higher doses, foci persisted for longer periods of time, consistent with the senescent phenotype [25,26]. Persistence of foci at 120 h was much less marked after 6 × 3 Gy than after a single dose of 18 Gy (Figure 1E-F).

### Reduced proliferative capacity and induction of cell senescence

Considering that a single dose of 18 Gy was able to induce a sustained DDR in CAF cultures, and that such dose is clinically relevant in the context of SART, its effect on proliferative capacity was assessed. Proliferation rates of CAFs from all donors were reduced after 18 Gy (Figure 2A). In parallel experiments, induction of cell senescence by β-galactosidase staining was measured six days after 18 Gy (single fraction) or 24 h after 6 daily fractions of 3 Gy (Figure 2B). Irradiated cultures showed prominent induction of β-galactosidase staining, suggesting that a large number of irradiated CAFs entered growth arrest by activating premature cell senescence mechanisms. The senescence response was more pronounced after 1 × 18 Gy than after 6 × 3 Gy (p < 0.05) (Figure 2C).

### Effects of AIR on migratory and invasive capacity

**Figure 3:**
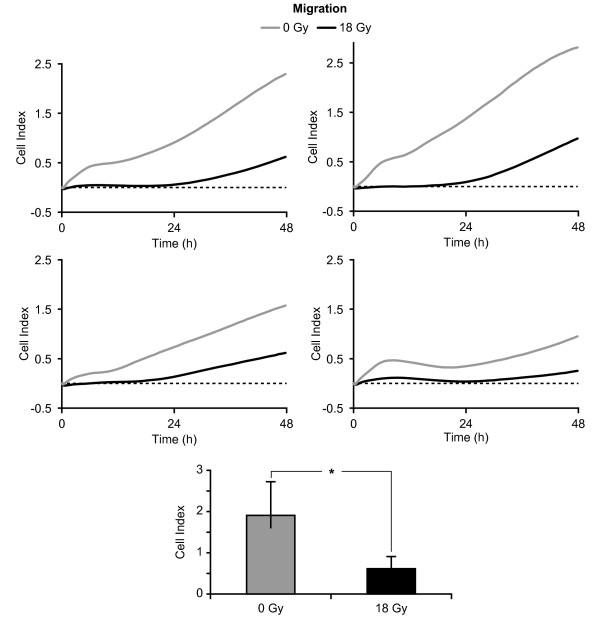
**Effects of AIR on the migration capacity of CAFs**. Migration curves of CAFs prepared from four randomly selected donors (donors #14, #6, #7, #8 in Table 1). Irradiated (black lines) and control (grey lines) CAFs were brought into suspension and loaded on CIM-plates, and the assay was run for 48 h. Columns in the lower panel represent average CI values at the end of the experiment from the four donors. Statistically significant differences found after applying paired sample *t-test *on values at the end point of the experiments are marked with * (p < 0.05).

**Figure 4:**
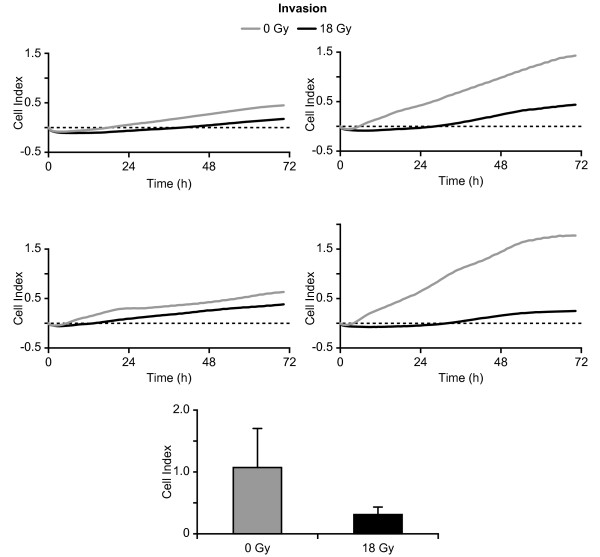
**Effects of AIR on the invasion capacity of CAFs**. Invasion curves of CAFs prepared from four randomly selected donors (donors #11, #2, #7, #13). Irradiated (black lines) and control (grey lines) CAFs were brought into suspension and loaded on CIM-plates coated with a Matrigel biofilm, and the assay was run for 72 h. Columns in the lower panel represent average CI values at the end of the experiment from the four donors. Statistical analyses by paired sampled *t*-test showed p = 0.09.

We went on to investigate the functional status of irradiated cells, with particular emphasis on invasion and migration, which have previously been shown to influence tumour invasiveness and angiogenesis. Irradiation of CAFs from all donors was associated with a significant drop in their migratory capacity (Figure 3). The migratory activity of irradiated cells was on average 67% (± 4.8) lower than non-irradiated cells when calculated 48 h after exposure to 1 × 18 Gy. The invasive capacity of irradiated CAFs across a biofilm of Matrigel was consistently abridged in all donors, averaging 66% (± 15.9%) reduction when compared with controls. However, the statistical analyses revealed not significant differences in values taken at the end point of the experiment (p = 0.09) (Figure 4). Overall, these results indicate that while CAFs appear to survive high radiation doses, their migratory and invasive capacity is considerably compromised.

### Effects of AIR on expression of ECM regulators

**Figure 5:**
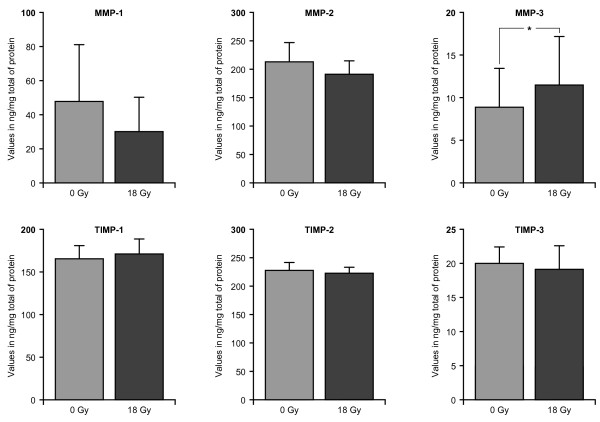
**Secretion of matrix metalloproteases and endogenous inhibitors by CAFs**. In panel (**A**), average determinations of the MMPs levels found in culture supernatants 4 to 6 days post-irradiation from 5 donors is represented (donors #1, #5, #7, #11, #14 in Table 1). Only MMPs that showed values above the detection limit of the assay are shown. Pale grey bars non-irradiated cultures; dark grey bars cells receiving (1 × 18 Gy). Statistically significant differences between control and irradiated cultures were observed for MMP-3 (p < 0.05), and in four out of five donors for MMP-1 (p = 0.06 all donors; p < 0.05 excluding donor-1). In panel (**B)**, expression patterns of TIMP-1, -2, -3 from the same supernatants used in (**A**) are shown.

After verifying that AIR reduces the invasive capacity of CAFs, we investigated potential mechanisms. Quantitative measurements of MMPs and their endogenous inhibitors, TIMPs, in culture supernatants were performed by multiplex protein detection assays (Figure 5A, B) in conditioned medium of CAFs from five donors. MMP-2 was the most abundantly expressed MMP, followed by MMP-1 and MMP-3 (Figure 5A). Expression levels of the latter two enzymes varied widely between CAFs from different donors. Conversely, MMP-7, MMP-8, MMP-9 and MMP-13 expression were below the detection limit of the assay (< 25 pg/ml) for both irradiated and non-irradiated CAFs (not shown). Comparative analyses showed that for 4 out of 5 donors, radiation of CAFs resulted in reduced secretion of MMP-1 (48% ± 23). No such reduction in MMP-1 secretion was observed in CAFs from a 5th donor (donor #7), but it is noteworthy that irradiation of CAFs from this donor caused a significant reduction in their migrative and invasive capacity (top right panel of Figures 3 and 4, respectively). This indicates that regulation of MMP-1 expression may play a minor or inconsistent role in regulating the invasive properties of CAFs. On the other hand, expression pattern for MMP-3 was modestly but consistently elevated following radiation in all five cell pairs (29% ± 17, p < 0.05). Regarding protease inhibitors, only TIMP-1, -2, and -3 could be detected in supernatants and no consistent alterations in their expression after AIR could be detected (Figure 5B). To assess the potential role played by the different MMPs on the invasive function of CAFs, the activity of CAFs-derived MMPs was abrogated by the exogenous administration of GM1489 (Calbiochem, Cat.# 364200, Merck KGaA, Darmstadt, Germany), a broad spectrum inhibitor of matrix metalloproteinases with high affinity for MMP-1 (Ki = 0.2 nM), and lower affinity for other MMPs (Ki = 500 nM for MMP2; Ki = 20 μM for MMP-3; Ki = 100 nM for MMP-8 and MMP-9). The invasion assays were carried out at increasing concentration of the inhibitor as indicated in Additional file 1: Figure S1A. At the lowest concentration of inhibitor (1 nM) more than 90% of MMP-1 activity should be blocked, however this amount if inhibitor exerted only 18% inhibition of invasion, whereas the invasion rates were reduced to approximately 50% at the highest concentration tested (10 μM) (Additional file 1: Figure S1B).

### Induction of cell surface integrin expression, cell adhesiveness and regulation of focal contacts

**Figure 6:**
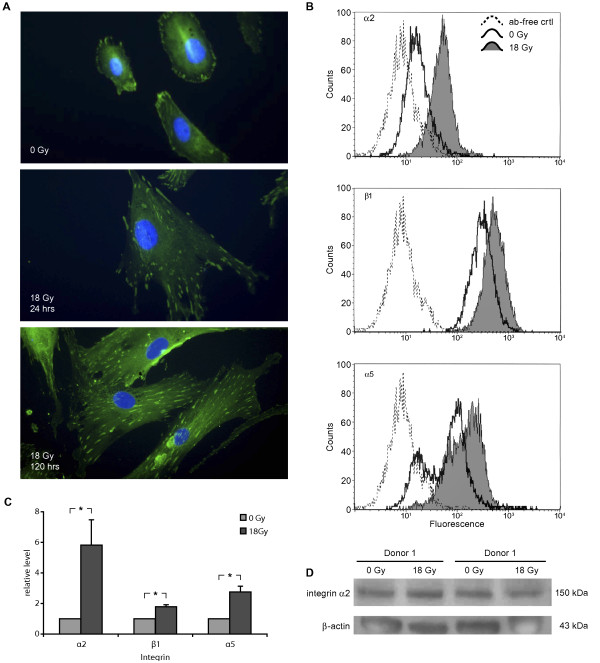
**Dynamics of focal contacts and changes in the expression of cell surface integrins**. Panels in (**A**) show AIR-induced changes of focal contacts by vinculin immunostaining, panels in (**B**) show FACS analyses of three different integrin subtypes (α2, β1, α5) in non-permeabilized CAFs from one representative donor (donor #1 in Table 1). In **(C)**, mean and standard error of the fluorescent intensities of control (pale grey) and irradiated (dark grey) CAFs from three randomly selected donors (donors #1, #5, and #14) is presented. Intensities at irradiated conditions are relative-values of the non-irradiated and normalized control values, indicating X-fold enhancement of cell surface expression after 1 × 18 Gy. Statistical significance was set at p < 0.05 by applying Student's paired *t*-test. In panel (**D**), the total cellular pool of integrin subunit α2 (~150 kDa) was analysed by Western blots in whole cell lysates from two randomly selected donors.

Last, we examined whether reduced motility of CAFs was linked to changes in expression of cell surface integrins and/or stabilization of focal contacts. Non-irradiated CAFs showed accumulation of vinculin in focal contacts distributed primarily at the periphery of cells (Figure 6A). Upon 1 × 18 Gy exposure the cellular distribution of focal contacts changed significantly: after 5 days CAFs showed a more flattened morphology and numerous focal contacts were distributed around the perimeter of cells. Furthermore, the surface expression of some ECM receptors was evaluated. Collagens and non-collagenous proteins such as laminin and fibronectin are abundantly expressed extracellular matrix components in the interstitial stroma of solid tumours. These matrix constituents serve as anchoring substrates for cells in tumours, thus directly affecting cell phenotype and dissemination [27]. In our study we have therefore chosen to study the expression of integrin subunits which are components of the ubiquitously expressed collagen receptor (integrin α2β1), and the fibronectin receptor (integrin α5β1). In pilot experiments, we show that the three integrin subunits selected for analyses all participate importantly to the migratory function of CAFs (Additional file 2: Figure S2). AIR provoked a donor-independent elevation in surface expression of all three integrins examined (α2, β1, α5) (Figure 6B). When values from three different donors were averaged, integrin α2 presented the most prominent response to AIR, with a 5.8-fold increase relative to controls, whereas integrins β1 and α5 were enhanced by factors of 1.8 and 2.7, respectively (Figure 6C). Expression levels of integrin α2 in whole cell lysates from two randomly selected donors (Figure 6D) demonstrated no difference between control and irradiated groups, indicating that changes were caused by redistribution of integrins rather than up-regulated expression.

## Discussion

This study was undertaken to shed light on the biological responses of cells from the stroma of lung tumours exposed to high radiation doses, proven to be successful in the treatment of medically inoperable NSCLCs. We show that ablative radiation doses exert therapeutically beneficial inhibitory effects on the proliferative, migratory and invasive capacity of CAFs, effects which are associated with increased focal adhesions, cell surface expression of integrins and modulation of MMP secretion.

Our initial experiments showed that radiation had a donor-independent inhibitory effect on the proliferative capacity of CAFs that was likely to be a consequence of radiation-induced senescence in a high proportion of cells. Ionizing radiation is well known to induce the same phenotype as replicative senescence, and is often referred to as stress-induced premature senescence (SIPS) [28]. In fibroblasts [13] and mesenchymal stem cells [29] a senescent phenotype typically develops over several days after exposure to IR. Such radiation-induced senescent fibroblasts have been postulated to retain metabolic function and to display tumour *promoting *effects through paracrine secretion of pro-inflammatory signals [13]. Furthermore, persistent DNA damage signalling has been linked to the establishment of a an irreversible senescent phenotype [13,25,26], and is also suggested as an indicator of lethal DNA damage [30]. On these premises we aimed to characterize the senescent phenotype of human CAFs and measure the kinetics of DNA damage foci several days post-irradiation. In our study, CAFs showed a progressive increase of β-galactosidase staining indicative of senescence, up to 5 days after AIR. Concomitantly, nuclear foci containing DNA-damage response elements were robustly activated by AIR and lasted several days, thus supporting the notion that a significant proportion of lung CAFs enter permanent senescence after exposure to a tumour ablative radiation dose.

Stromal fibroblasts are considered to be the master regulators of matrix remodelling [5,6]. We explored the influence of AIR on secretion by CAFs of key matrix regulators including several MMPs and their endogenous inhibitors; TIMPs. Our data show that only MMP-1 (collagenase), MMP-2 (gelatinase-A) and MMP-3 (stromelysin-1) are substantially secreted by lung CAFs. On the contrary, MMP-7, MMP-8, MMP-9 and MMP-13 were undetectable within the limits of the assay. We have observed by various means that MMP-9 is not significantly expressed by neither irradiated nor control lung CAFs, contributing to the notion that MMP-9 is primarily expressed by tumour-infiltrating inflammatory cells, rather than by CAFs [31].

A clear variation in expression among donors could be observed for MMP-1 and MMP-3 at both early and late time points after radiation. It remains to be explored if inter-individual variation in the inherent expression of these MMPs by CAFs has any impact on the overall tumour response to radiation among patients. Radiation was associated with changes in expression of MMP-1 and MMP-3, when examined 4 to 6 days after treatment. Secreted MMP-1 protein-levels were significantly reduced in 4 out of 5 cases, whereas MMP-3 levels were enhanced in irradiated CAFs from all donors included in the experiments. Patients with tumours expressing MMP-1 at the primary site are reported to have a significantly worse prognosis than MMP-1 negative patients [32]. The AIR-mediated reduction of MMP-1 expression could, in part, explain the repressed invasiveness of CAFs. To rule out this hypothesis we tested the invasive capacity of CAFs in the presence of an MMP inhibitor, GM1489 [33] (Additional file 1: figure S1). Our data show that at a concentration of 1 nM of GM1489, an amount that would inhibit over 90% of MMP-1 activity and less than 1% of other MMPs, the rate of invasion is reduced only 18%. These results indicate that MMP-1 only play a modest role for CAF invasion, at least as observed in our *in vitro *assay, and its reduced expression may not account for the reduced invasion observed.

On the contrary, enhanced levels of MMP-3 might represent a negative impact of AIR-based therapy, since secreted MMP-3 has been reported to correlate with tumorigenicity and invasiveness [34]. However, recent studies indicate that MMP-3, as well as other MMPs, may also have tumour-suppressive effects [35]. When considering the catalytic activity and quantities of MMPs, it must be taken into account that these proteases are highly regulated at multiple levels, including transcription, secretion, activation of the inactive pro-enzymes, and finally, the counterbalancing effect mediated by TIMPs [36]. We also found that TIMP-1, TIMP-2 and to a lesser extent TIMP-3 are actively secreted by lung CAFs. However, radiation exposure did not mediate consistent stimulatory or inhibitory effects on the TIMP levels.

CAF motility is a fundamental function supporting tumour growth, invasiveness and angiogenesis. Cell adhesion to ECM and locomotion is mediated by cell surface receptors called integrins, whose ECM ligand specificity is determined by combinations of α and β integrin subunits [37,38]. Integrins expressed on stromal fibroblasts contribute significantly in the regulation of tumour development and metastasis by affecting the migratory capacity of fibroblasts, by regulating cell proliferation and survival, and by modifying growth factor signalling [37]. In our study, we show a dramatic redistribution of focal contacts upon AIR that appears concomitantly with the acquirement of the senescent phenotype. On the other hand, flow cytometric analysis clearly demonstrated a radiation-induced increase in surface expression of the integrin subunits examined (α2, β1, α5). Expression levels in whole cell lysates further revealed that the total protein pool of integrin α2 was unaffected after exposure to 18 Gy, suggesting that the surface accumulation was likely to be a consequence of reduced internalization and/or enhanced recycling of integrins from the intracellular pool [39]. Interestingly, a similar enhancement (2-fold) of β1-integrin surface levels was recently demonstrated in cancer cells with defective endocytic machinery [40].

Overall, our findings support the view that stabilization of focal contacts (via integrins) increases attachment and impairs migration of CAFs [41]. Radiation-induced enhancement of cell adhesion has also been demonstrated by Cordes and co-workers in various cell types, and was reflected by increased cell-surface expression of β1-integrin [42]. In fact, augmentation of cell surface expression of integrins, in particular β1-integrin, has been postulated as a cellular mechanism to potentiate anchorage-dependent pro-survival anti-apoptotic pathways through binding to ECM components [43]. Furthermore, inhibition of β1-integrin reportedly mediate enhanced radioresponse, and has been suggested as a therapeutic target [44].

## Conclusions

Our data show that ablative radiation doses exert advantageous inhibitory effects on the proliferative, migratory and invasive capacity on CAFs, thus hindering some of their pro-malignant properties. Reduction of MMP-1 expression and enhanced accumulation of integrins at the cell surface are two key phenotypic changes induced by AIR that might explain the reduced motility of CAFs. The downstream consequences of these phenotypic changes on the overall tumour response to AIR remain to be elucidated. The interpretation of our results however needs some cautiousness since the AIR-induced changes on CAFs' phenotype and functions that we describe in this study could be somehow transformed in the presence of lung cancer cells or other stromal cells. Thus, the ultimate impact of irradiated fibroblasts on tumour development still needs to be explored in more complex *in vivo *settings.

## Abbreviations

IR: Ionizing radiation; RT: Radiation therapy; AIR: Ablative ionizing radiation; CAF: Cancer-associated fibroblast; MMP: Matrix metalloproteinase; TIMP: Tissue inhibitor of metalloproteinases; ECM: Extracellular matrix.

## Competing interests

The authors declare that they have no competing interests.

## Authors' contributions

TH and IM conceived the study. TH was responsible for establishment and performance of the radiation protocols, evaluation of all data and writing of the manuscript. IM planned the overall experimental strategy, carried out most of the experimental work, and shared with TH the main part of the writing. IP contributed in the establishment and characterization of cells, senescence assays and the western blot analyses. VB performed the measurements of MMPs and TIMPs, prepared the graphs, performed the statistics and helped to revise the manuscript. BTM was responsible for running all flow cytometry analysis and helped in interpreting results. JOW contributed in the planning evaluation and interpretation of work related to MMPs and TIMPs. KB provided tumour tissue and contributed to drafting the manuscript. LTB had responsibility for pathological diagnostics and handling of fresh human tumour specimens. AC and RHP participated in the overall interpretation of data and helped to draft the manuscript. RB participated in the overall coordination of the study and proof-reading of the manuscript.

## Supplementary Material

Additional file 1 **Figure S1 *Role of MMPs on the invasive capacity by CAFs***. The activity of CAFs-derived MMPs was abrogated by the exogenous administration of GM1489, a broad spectrum inhibitor of matrix metalloproteinases with high affinity for MMP-1 (Ki = 0.2 nM). The invasion assays were carried out at increasing concentration of the inhibitor as indicated in Figure (A). At the lowest concentration of inhibitor (1 nM) more than 90% of MMP-1 activity should be blocked, however this amount if inhibitor exerted only 18% inhibition of invasion, whereas the invasion rates were reduced to approximately 50% at the highest concentration tested (10 μM) (B). Click here for file

Additional file 2 **Figure S2 *Role of integrin β1, α2 and α5 on the migratory function of CAFs***. The participation of each integrin subunit on the migratory capacity of CAFs was checked by measuring CAF migration rates in the presence of specific integrin antibodies. Cells were incubated with10 μg/ml of each antibody for 30 min at room temperature before the assay and then tested for migration in the continued presence of the antibodies. Wide-spectrum inhibition of integrin binding was checked by administration of 100 μM RGD and control peptides. All treatments, except control peptides and IgG, were able to reduce extensively and permanently the migratory capacity of CAFs. Click here for file
