# Supplementary figures and images for: Cancer-associated fibroblasts from human NSCLC survive ablative doses of radiation but their invasive capacity is reduced
Source: Radiat Oncol. 2012 Apr 13;7:59. doi: 10.1186/1748-717X-7-59 (PMC3359264; doi:10.1186/1748-717X-7-59)

## Slide 1
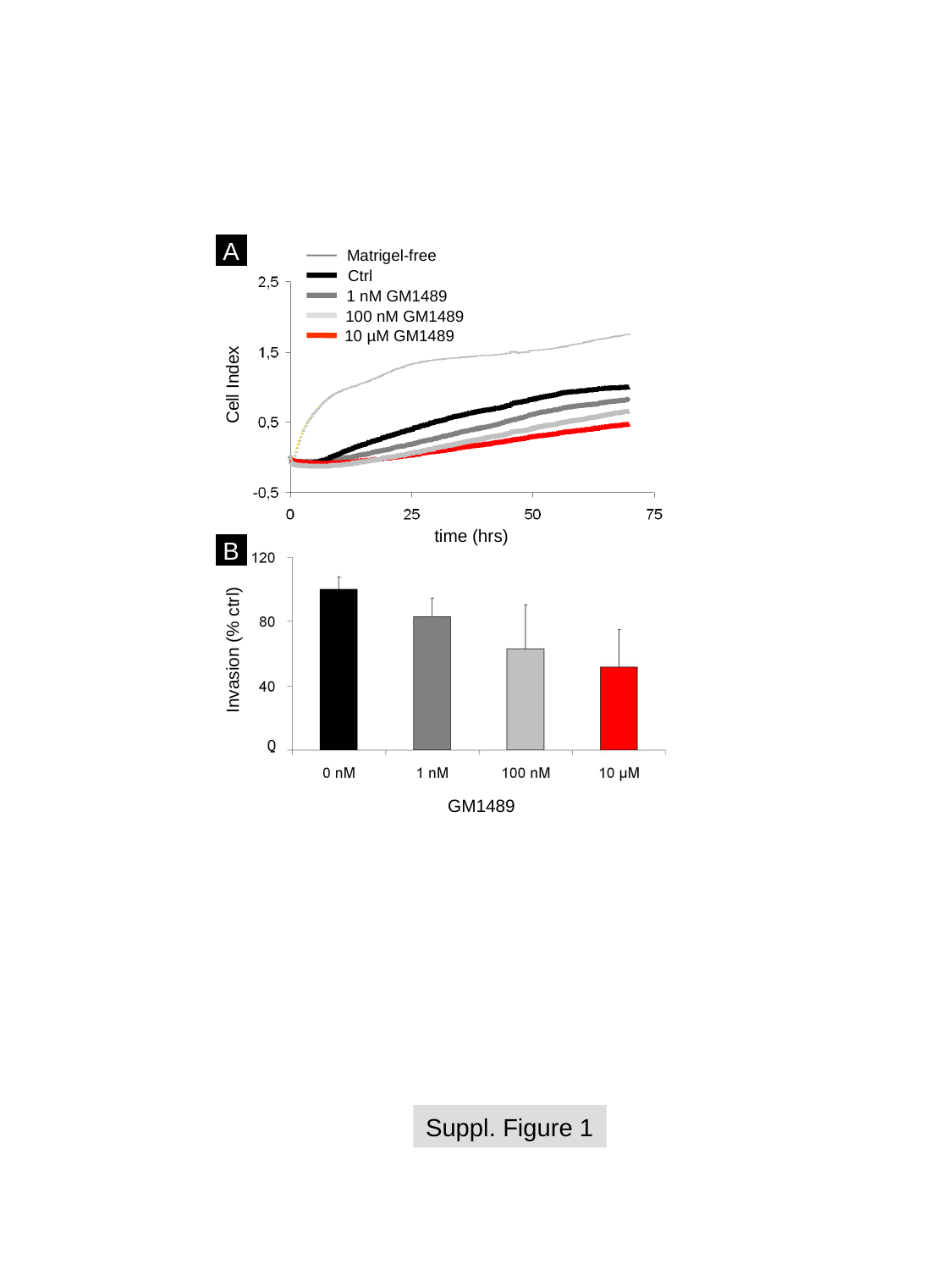

A
Matrigel-free
Ctrl
1 nM GM1489
2.0
100 nM GM1489
10 µM GM1489
1.0
Cell Index
0.0
0
20
40
60
time (hrs)
B
Invasion (% ctrl)
0
GM1489
Suppl. Figure 1

Supplement: Additional file 1 — Figure S1 Role of MMPs on the invasive capacity by CAFs. The activity of CAFs-derived MMPs was abrogated by the exogenous administration of GM1489, a broad spectrum inhibitor of matrix metalloproteinases with high affinity for MMP-1 (Ki = 0.2 nM). The invasion assays were carried out at increasing concentration of the inhibitor as indicated in Figure (A). At the lowest concentration of inhibitor (1 nM) more than 90% of MMP-1 activity should be blocked, however this amount if inhibitor exerted only 18% inhibition of invasion, whereas the invasion rates were reduced to approximately 50% at the highest concentration tested (10 μM) (B). [file 1748-717X-7-59-S1.PPT]
