# Supplementary material for: Cancer-associated fibroblasts from human NSCLC survive ablative doses of radiation but their invasive capacity is reduced
Source: Radiat Oncol. 2012 Apr 13;7:59. doi: 10.1186/1748-717X-7-59 (PMC3359264; doi:10.1186/1748-717X-7-59)
Supplement: Additional file 2 — Figure S2 Role of integrin β1, α2 and α5 on the migratory function of CAFs. The participation of each integrin subunit on the migratory capacity of CAFs was checked by measuring CAF migration rates in the presence of specific integrin antibodies. Cells were incubated with10 μg/ml of each antibody for 30 min at room temperature before the assay and then tested for migration in the continued presence of the antibodies. Wide-spectrum inhibition of integrin binding was checked by administration of 100 μM RGD and control peptides. All treatments, except control peptides and IgG, were able to reduce extensively and permanently the migratory capacity of CAFs. [file 1748-717X-7-59-S2.PPT]

## Slide 1
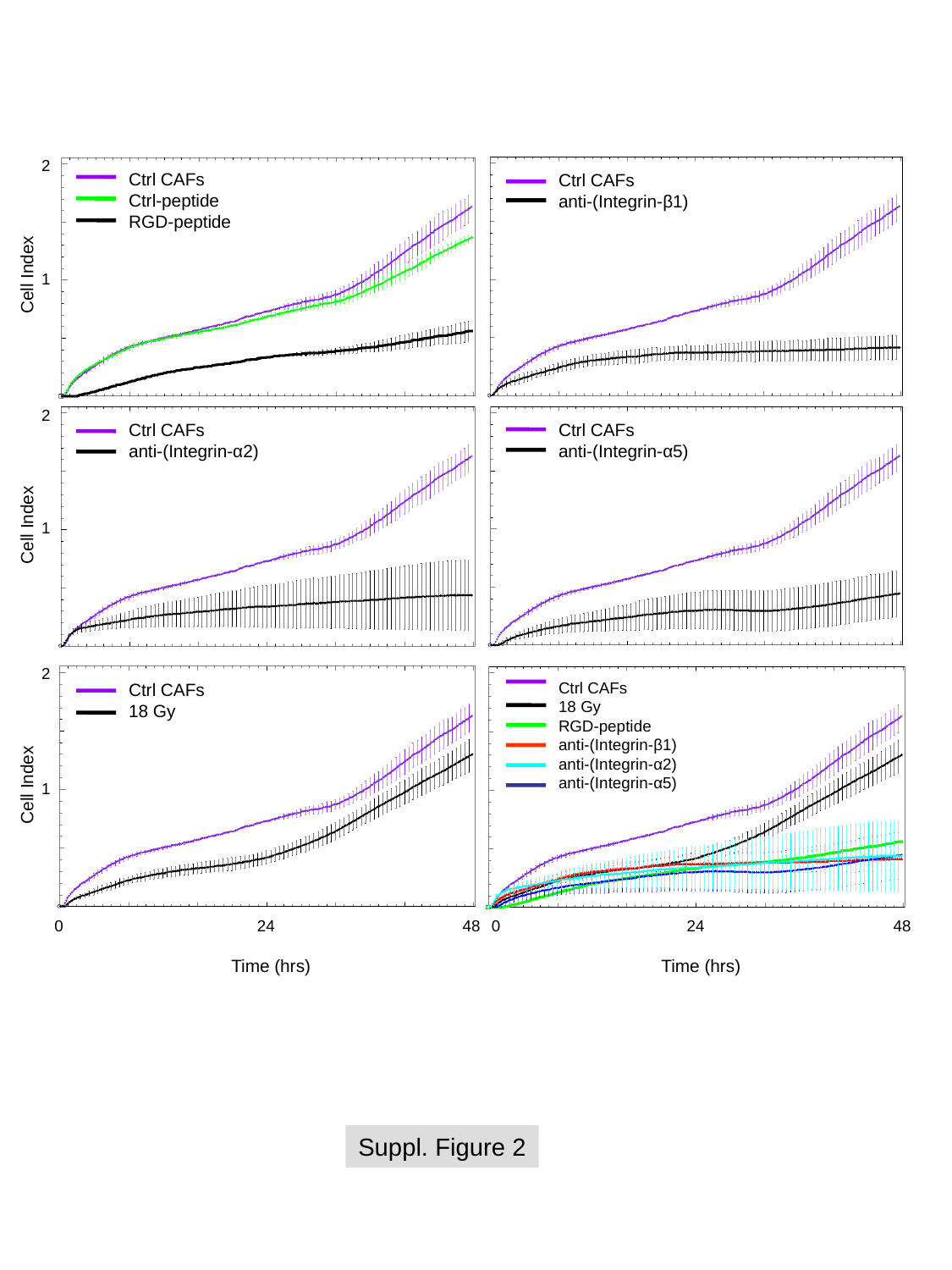

2
Ctrl CAFs
Ctrl-peptide
RGD-peptide
Ctrl CAFs
anti-(Integrin-β1)
Cell Index
1
2
Ctrl CAFs
anti-(Integrin-α2)
Ctrl CAFs
anti-(Integrin-α5)
Cell Index
1
2
Ctrl CAFs
18 Gy
RGD-peptide
anti-(Integrin-β1)
anti-(Integrin-α2)
anti-(Integrin-α5)
Ctrl CAFs
18 Gy
Cell Index
1
0
24
48
0
24
48
Time (hrs)
Time (hrs)
Suppl. Figure 2
